# An unbiased approach to identify genes involved in development in a turtle with temperature-dependent sex determination

**DOI:** 10.1186/1471-2164-13-308

**Published:** 2012-07-15

**Authors:** Jena L Chojnowski, Edward L Braun

**Affiliations:** 1 Genetics Department, University of Georgia, 500 DW Brooks Dr., Coverdell Center Rm270, Athens, GA, 30602, USA; 2 Department of Biology, University of Florida, PO Box 118525, Gainesville, FL, 32607, USA

## Abstract

**Background:**

Many reptiles exhibit temperature-dependent sex determination (TSD). The initial cue in TSD is incubation temperature, unlike genotypic sex determination (GSD) where it is determined by the presence of specific alleles (or genetic loci). We used patterns of gene expression to identify candidates for genes with a role in TSD and other developmental processes without making *a priori* assumptions about the identity of these genes (ortholog-based approach). We identified genes with sexually dimorphic mRNA accumulation during the temperature sensitive period of development in the Red-eared slider turtle (*Trachemys scripta*), a turtle with TSD. Genes with differential mRNA accumulation in response to estrogen (estradiol-17β; E_2_) exposure and developmental stages were also identified.

**Results:**

Sequencing 767 clones from three suppression-subtractive hybridization libraries yielded a total of 581 unique sequences. Screening a macroarray with a subset of those sequences revealed a total of 26 genes that exhibited differential mRNA accumulation: 16 female biased and 10 male biased. Additional analyses revealed that *C16ORF62* (an unknown gene) and *MALAT1* (a long noncoding RNA) exhibited increased mRNA accumulation at the male producing temperature relative to the female producing temperature during embryonic sexual development. Finally, we identified four genes (*C16ORF62, CCT3, MMP2*, and *NFIB*) that exhibited a stage effect and five genes (*C16ORF62, CCT3, MMP2, NFIB* and *NOTCH2*) showed a response to E_2_ exposure.

**Conclusions:**

Here we report a survey of genes identified using patterns of mRNA accumulation during embryonic development in a turtle with TSD. Many previous studies have focused on examining the turtle orthologs of genes involved in mammalian development. Although valuable, the limitations of this approach are exemplified by our identification of two genes (*MALAT1* and *C16ORF62*) that are sexually dimorphic during embryonic development. *MALAT1* is a noncoding RNA that has not been implicated in sexual differentiation in other vertebrates and *C16ORF62* has an unknown function. Our results revealed genes that are candidates for having roles in turtle embryonic development, including TSD, and highlight the need to expand our search parameters beyond protein-coding genes.

## Background

Turtles have been characterized for a small number of fascinating differences from the better-studied groups of amniotes, like the regulation of sexual development by temperature and the presence of the carapace. However, little is known about genes involved in the many aspects of turtle development. The development of the eye [1,2], brain [3,4], carapace [reviewed in [5]], and gonads (Table 1); and the role of specific hormones [reviewed in [6]] have all been briefly studied but these studies have focused on the orthologs of genes already known to have a role in human and mouse development. Although the use of studies focused on the turtle orthologs of genes identified in other organisms are clearly important, screens for candidate genes that avoid making *a priori* assumptions represent a complementary approach with excellent potential to reveal novel developmental genes. This study uses such an approach to identify genes with sexually dimorphic expression; this set of genes is enriched for genes involved in temperature-dependent sex determination (TSD), but will also include genes in other aspects of sexual differentiation and general developmental processes related to temperature and hormone regulation that might not be directly related to sexual differentiation.

**Table 1:** A general overview of sexually dimorphic gene expression in turtles with TSD

	**stage 17**		**late in TSP**				
Gene	Testis	Ovary	Testis	Ovary	Testis	Ovary	Reference
*SF1*	+		+		+		[7,8]
*WT1*	same	same	same	same	same	same	[9-11]
*DAX1*	same	same	same	same	same	same	[12]
*SOX9*	same	same	+		+		[11,13]
*DMRT1*	+		+		+		[14-16]
*CYP19*	same	same		+		+	[8,15,17]
*SOX8*	same	same	same	same	same	same	[18]
*FOXL2*	same	same		+		+	[12]
*MIS*	same	same	+		+		[8,18]
*R-SPONDIN*	same	same		+		+	[19]
*WNT4*	same	same	same	same		+	[12]

Many reptilian taxa, including the majority of turtle species, exhibit TSD [20,21]. Incubation temperature is the initial cue for sexual development in TSD, in contrast to genetic sex determination (GSD) that is evident in a number of vertebrate groups such as amphibians, snakes, birds, and mammals [22]. GSD is best characterized in therian mammals and is initiated by the *SRY* gene, located on the Y chromosome, which causes organisms expressing the gene to develop as males [23]. *SRY* orthologs have not been identified in other groups of vertebrates regardless of whether they exhibit TSD or GSD, suggesting that *SRY* is an innovation unique to therian mammals. In fact, only one other unique “trigger” gene for sexual development has been identified in a vertebrate taxon, a fish with GSD (medaka; see [24,25]). It is unclear whether a trigger gene exists in organisms that exhibit TSD, since there are several models that can explain TSD. For example, TSD may reflect regulation of a trigger gene (or set of trigger genes) by incubation temperature, it may reflect the impact of temperature upon the activity of specific enzymes that have a role in signaling, or it may reflect a combination of both phenomena [9,12,13]. Regardless, it is clear based upon the studies in organisms with known triggers that a gene homologous to a known trigger in other organisms does not regulate TSD in turtles [26].

Although trigger gene(s) are not conserved, if one or more are even present, a number of genes involved in gonadal differentiation and other aspects of sexual development are conserved among vertebrates, including organisms with different sex determining systems [27,28]. A number of orthologs of genes first identified in mammals have been identified and characterized in different vertebrate groups [29-33], including turtles (Table 1). Studies focused on orthologs of genes known to play a role in mammalian sexual development have provided valuable information, although this approach has limits for broader investigations. One setback to an ortholog approach is that the complete set of genes involved in mammalian GSD remains unknown and therefore, it is restricting in finding novel genes or pathways specific to a new system. A complementary approach is to identify candidate genes using patterns of expression rather than orthology. This raises the question of the most appropriate tissue to assay gene expression, since sexually dimorphic gene expression has been noted in multiple tissues [13,34-36]. Thus, focusing on specific tissues may result in the omission of critical candidate genes. The use of whole embryos is an unbiased strategy for finding candidate genes for sex determination, but it also allows for the identification of genes in other pathways that occur within the same time points. Also, temperature and hormones do not exclusively affect sex determination, and therefore novel genes can also be identified that are related to temperature and the effects of hormonal exposure.

The goal of this study is to use the red-eared slider turtle (*Trachemys scripta*), a turtle with TSD, to identify candidate genes within the embryonic development pathway. Genes that exhibited a sexually dimorphic expression pattern during the temperature-sensitive period (TSP), which contains the critical stage for commitment to a specific sex [37], were targeted. The approach we used will also reveal genes that exhibit a differential expression pattern due to temperature or hormonal differences that are not directly related to sexual development. To accomplish this search we identified genes that show increased mRNA accumulation under either the male or female producing temperatures as well as genes that exhibit response to estrogen exposure. Since we also examined embryos at various times during the TSP it was also possible to identify stage effects upon mRNA accumulation during the TSP. These different searches allow for a broader investigation for not only sexually specific factors but also for general developmental genes previously unknown within the time points of the TSP. To accomplish this, we produced three subtraction libraries. Two of these libraries were enriched for genes that show higher mRNA accumulation during the TSP in one specific temperature regime (i.e., genes that exhibit greater mRNA accumulation at the female-producing temperature [31°C] than at the male-producing temperature [26°C] and vice versa). The third library was enriched for genes that show increased mRNA accumulation during the TSP in embryos produced at the male-producing temperature after treatment with exogenous estrogen, ultimately producing the female phenotype. Subsets of the cDNAs from these libraries were examined more thoroughly by macroarray hybridization and semi-quantitative PCR or quantitative real-time PCR. This approach has the potential to identify developmental genes that exhibit differential expression when red-eared slider turtles are exposed to different temperatures and hormonal conditions during the TSP without making *a priori* assumptions about the identity of the genes.

## Results and discussion

### Suppression subtraction hybridization (SSH) libraries

SSH was used to construct libraries enriched for cDNAs that correspond to mRNAs that exhibit different levels of accumulation during the TSP. Three subtracted cDNA libraries were constructed: one enriched for mRNAs that accumulate at higher levels at the female-producing temperature than the male-producing temperature (hereafter called the “female library”); another enriched for mRNAs that accumulate at higher levels at the male-producing temperature than the female-producing temperature (hereafter called the “male library”); and a third enriched for mRNAs that accumulate at higher levels at the male-producing temperature with exogenous estradiol-17β (sufficient for sex reversal) than in similar embryos treated with the vehicle alone (hereafter called the “E_2_ library”). A total of 767 sequences were obtained and were previously deposited in dbEST (FG341000:FG341832). The SSH expressed sequence tags (ESTs) were processed as described [38,39], yielding a total of 581 contigs and singletons (unigenes) after assembly using CAP3 [40]. The results for the homology searches can be found in Additional file 1: Homology search for subtraction libraries.

SSH libraries typically contain some housekeeping genes [41-43] since it is difficult to completely eliminate genes that do not exhibit differential expression between the two experimental conditions. The quality of SSH libraries can be assessed by examining the proportion of broadly expressed genes, although the most appropriate sets of genes to view as “housekeeping” can be problematic to define precisely [44]. However, analyses of GC-content [39] provide a line of evidence that the proportion of cDNAs that correspond to housekeeping gene transcripts is greatly reduced in our SSH libraries; housekeeping genes tend to have a higher GC-content than genes that exhibit lower levels of expression [45,46] and it provides an efficient and practical method to define housekeeping genes that avoids conflicts among the available lists of housekeeping genes. We found that the GC-content of the turtle transcripts was lower than expected for other reptilian EST efforts [39]. Thus, the SSH method did appear to enrich for genes with lower levels of mRNA accumulation despite being unable, as expected, to eliminate all housekeeping gene cDNAs.

### Genes found in the SSH libraries

GeneMerge was used to test for over-represented GO (Gene Ontology) terms signifying biological processes from genes with human homologs found in all three SSH libraries [47] (Additional file 2: GeneMerge for biological processes). A total of 34 over-represented GO terms were significant (p < 0.05) and they represent a broad range of biological processes. A few umbrella categories that include a number of over-represented GO terms are anatomical structure morphogenesis (GO:0009653; includes face morphogenesis [GO:0060325] and skeletal system morphogenesis [GO:0048705]), cellular processing (GO:0009987; includes ribosomal small subunit biogenesis [GO:0042274], T cell differentiation in the thymus [GO:0033077], cellular membrane organization [GO:0016044], DNA packaging [GO:0006323], regulation of cell cycle [GO:0051726], negative regulation of apoptosis [GO:0043066]), and metabolic processing (GO:0044267; includes translation [GO:0006412], transcription [GO:0006350], protein folding [GO:0006457], translational initiation [GO:0006413], and translational elongation [GO:0006414]). These categories show that the genes found in the SSH libraries involve active cell differentiation and processing, as expected for mRNAs expressed in developing embryos.

**Figure 1:**
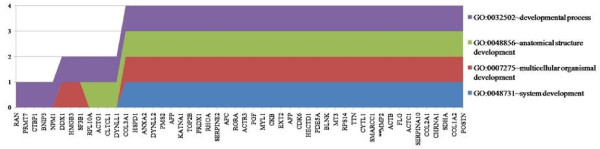
**Development Cluster from DAVID functional annotation clustering with high stringency.** Functional cluster of developmental genes and GO terms from the resulting known genes from the SSH libraries with high stringency.

In addition, genes that have human homologs from the SSH libraries were clustered into functionally related groups within the subset of biological processes by the DAVID tool for functional annotation clustering with high stringency [48,49] (Additional file 3: DAVID functional annotation clustering). The different groups represent the diversity of the libraries’ genes. A pertinent cluster to our study that emerged is one enriched for genes involved in developmental processes (Figure 1). Though these genes are identified as being associated with human developmental processes, this study offers a chance to determine if they have been co-opted for similar functions in the turtle. One of the genes from this cluster *MMP2*, starred in Figure 1, is of particular interest because it is one of the first *MIS* (Müllerian inhibiting substance)-target genes involved in Müllerian duct regression and is involved in the breakdown of extracellular matrix in normal physiological processes, such as embryonic development, reproduction, and tissue remodelling [50]. *MMP2*’s involvement in mammalian development leads us to believe it has potential to be a candidate gene for relevant to the aspects of turtle development that represented the focus of this study, potentially including TSD.

A number of distinct genes (7) in the SSH libraries encode temperature responsive proteins or regulatory genes involved in the heat shock response. Ten temperature responsive cDNAs were found in the female library (two of which exhibited within-library redundancy) whereas only one of each was found in the male and E_2_ libraries (Table 2). Since the female library was enriched for genes expressed at a temperature 5°C higher than either the male or the E_2_ libraries the larger number of heat shock cDNAs could simply reflect a temperature effect. However, specific temperature responsive mRNAs accumulate differentially during gonadal differentiation in another reptile with TSD (*Alligator mississippiensis*; [51]). Furthermore, specific heat shock proteins play a critical role in the transcriptional complex of steroid hormone receptors and their corresponding chaperones and cofactors [52]. Given that temperature is the initial signal in TSD, temperature responsive genes represent good candidates for involvement in the TSD cascade.

**Table 2:** Temperature responsive genes found in SSH

**Name**	**Library where found**	**Redundancy within library**
*HSPA8*	Male	1
*HSP90B1*	E_2_	1
*CIRBP*	Female	2
*HSBP1*	Female	3
*HSP90AA1*	Female	1
*HSPD1*	Female	1
*SERPINH1*	Female	1

### Differential expression revealed by macroarray analyses

**Figure 2:**
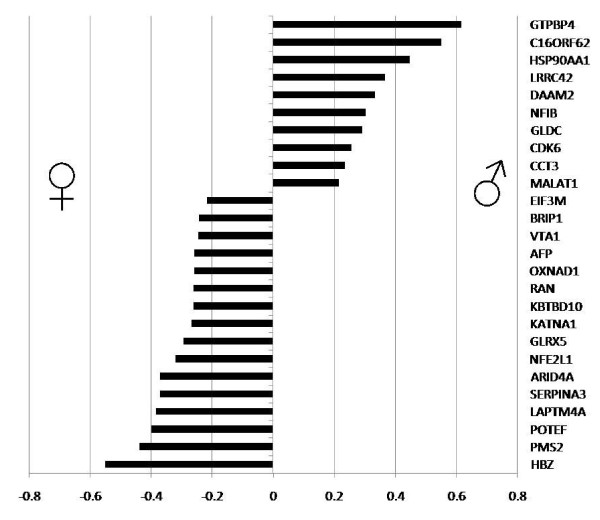
**Macroarray results showing sexually dimorphic expression patterns.** Log view of the fold change between female and male expression patterns determined from a macroarray.

A macroarray assay was used to refine the set of genes identified by sequencing the SSH libraries for sexual dimorphism and place our analysis of transcript accumulation under different experimental conditions in a quantitative framework (Figure 2). A total of 26 signals were detected as having differential expression patterns: 16 female biased signals and 10 male biased signals. However, the degree of differential expression revealed by the macroarray analyses was typically <2-fold. Thus, our macroarray analyses were able to show that a number of cDNAs present in the SSH libraries do exhibit sexual dimorphic patterns of expression under the conditions we tested, although the differences in the amount of mRNA present was typically limited. Since our experiments were conducted on whole embryos to ensure unbiased candidate gene identification, it might be the case that some genes identified exhibit stronger sexual dimorphism in a specific tissue or a subset of tissues (e.g. brain [34,35], liver [36], and gonad [13]). However, our results indicate that sexually dimorphic gene expression is detectable at the whole embryo level, indicating that the unbiased approach is feasible.

Genes that are found to be sexually dimorphic are not automatically considered to be part of TSD since differing temperatures and hormones can affect more than just sexual development. The genes that emerged from the macroarray as being differentially expressed have a mixture of biological roles in humans based on DAVID. Some genes overlap in their biological roles while others have more distinct roles. For example, 10 genes (*GTPBP4, HSP90AA1, ARID4A, RAN, HBZ, SERPINA3, BRIP1, NFE2L1, CDK6*, and *NFIB*) are involved in the regulation of metabolic processing and 6 genes (*GTPBP4, BRIP1, RAN, KATNA1, CDK6*, and *NFIB*) are involved in cell division and proliferation (5 of the 6 in the later categories are also found in the earlier category of regulation of metabolic processing). Moreover, *AFP* and *LAPTM4A* are involved in reproduction and transport, respectively. Though these genes are not directly related to human sexual development, they provide us with a clue as to which types of processes occur during turtle development under different temperatures and hormone exposures.

### Semi quantitative (semiQ) PCR validation of TSD candidate genes

**Figure 3:**
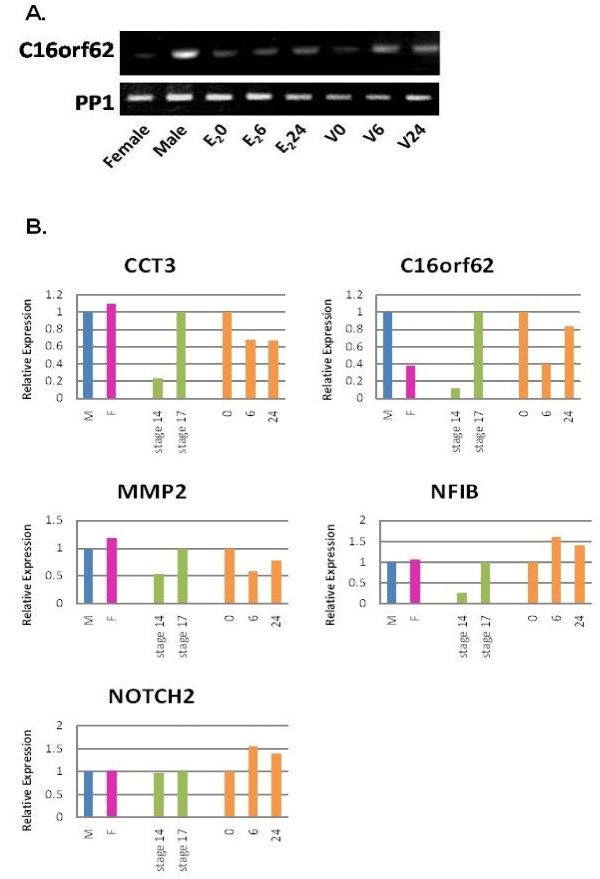
**Semi-quantitative PCR Results.** A. An example of a semi-quantitative gel image. B. Semi-quantitative results for sexual dimorphism (M = male, F = female), stage effect between males (green bars), and an E_2_ time trial (orange bars).

Further experiments for validation were conducted on a subset of candidate genes selected from both the subtraction library and macroarray analyses. These genes were chosen because they were implicated in mammalian sexual development (*MMP2*: [53]), mammalian development (*CCT3, NFIB,* and *NOTCH2*[54-56]), or have an unknown function (*C16ORF62*). The five candidate genes were used in three different categories for semiQ-PCR: sexual dimorphic expression at stage 17, differences between stages 14 and 17, and an E_2_ time trial conducted during stage 14 including fast (6 hours) and slow (24 hours) responses (Figure 3).

Only the turtle ortholog of *C16ORF62*, a gene of unknown function, showed evidence of sexually dimorphic expression at stage 17. The transcript of this gene showed greater accumulation in males than in females (~2.5 fold increase), the same trend that was evident in the macroarray results. It is conserved in mammals, birds, fish, insects, nematodes, and plants, its GO term is Integral to membrane, and it is found in a wide variety of adult and embryonic tissues in mammals [57,58]. Since *C16ORF62* is a gene of unknown function and it was found to be sexually dimorphic in turtle embryos during the TSP, it represents truly a novel candidate for a gene involved in turtle development, potentially including TSD; and potentially in sexual development in other vertebrates as well.

Four genes exhibited increased accumulation during stage 17 relative to stage 14 in embryos incubated at the male producing temperature (26°C). This stage effect was evident for *C16ORF62, CCT3, MMP2*, and *NFIB* (Figure 3B); the most striking is a ~8-fold increase in mRNA accumulation between stage 14 and 17 for *C16ORF62*. The others showed a range of relative increase in mRNA accumulation of 1.2-fold to 4.8-fold with stage progression. *CCT3, MMP2*, and *NFIB* have been implicated in gonad development (*MMP2*: [59]) or other aspects of development (*NFIB* and *CCT3*: [54,55]) in mammals. When this information is combined with our observation that turtle orthologs exhibited increased mRNA accumulation as development proceeded from stage 14 to stage 17 (early in TSP) it is reasonable to speculate that these genes play a role in turtle development, potentially sexual development in the case of *MMP2*. Though *NOTCH2* does not show a stage effect for turtle it has been previously seen as a developmental gene in neuronal development of a mammal and might have a more significant affect on turtle development during different stages or the stage differences are too low to identify with this study [56].

All five genes show a rapid (6 hours) response to E_2_ exposure. *C16ORF62, CCT3,* and *MMP2* all show a downregulation of mRNA expression and *NFIB* and *NOTCH2* show an upregulation. Four of the genes (*CCT3, MMP2NFIB*, and *NOTCH2*) exhibited similar mRNA accumulation both 6 hours and 24 hours after E_2_ exposure; accumulation of the *C16ORF62* mRNA almost returned to pre-exposure levels after 24 hours. *MMP2* is affected by E_2_ based upon previous studies whereas the impact of E_2_ exposure upon mRNA accumulation for other genes has not been examined. Mahmoodzadeh et al. [59] showed that E_2_ inhibits *MMP2* gene expression in rat fibroblasts and those results corroborate our findings for *MMP2*’s involvement with E_2_.

### Expression of a long noncoding RNA (ncRNA) is sexually-dimorphic

A number of cDNAs on the macroarray (61) could not be identified using BLASTX, suggesting that they correspond either to cDNAs for which only untranslated region was included in the EST read or noncoding RNAs (ncRNA). To identify some of these cDNAs we conducted BLASTN searches and revealed that one of the cDNAs that exhibits sexual dimorphism is a ncRNA, *MALAT1*.

*MALAT1* is a long (~7 kb) ncRNA that undergoes a cleavage that produces two RNAs, a smaller tRNA-like cytoplasmic RNA (~61nt) and a 6.7 kb RNA that localize to two different subcellular compartments, cytoplasm and nuclear speckles respectively [60]. Characteristically, it has short blocks of high conservation across the entire transcript, especially in 3’ half of RNA, and lacks repetitive elements except for a SINE and LINE element near its 5’ end [60]. The smaller (~61nt) transcript generated by cleavage is highly conserved across many species, including mouse, human, dog, lizard, frog, and stickleback. It has not yet been found in any of the four available sequenced bird genomes (chicken, turkey, zebrafinch, and duck). There are two possible explanations: (1) birds lost the locus or (2) it is located in a region that was consistently underrepresented in all of the available avian genome assemblies [61-64]. Both explanations are plausible since gene loss is known to be an important process during evolution [65] but the avian genome assemblies (like other vertebrate genome assemblies) are incomplete.

*MALAT1* shows a broad distribution of expression in normal human and mouse tissues but its misregulation is correlated with the progression of cancers and it is upregulated in many human carcinomas [66-69]. More importantly for this study, *MALAT1* accumulation is higher in adult mammalian ovaries than adult testes [60,70]. However, the pattern of differential expression for *MALAT1* in adult mammalian gonads is distinct from the pattern we observed using the macroarray assay, in which the mRNA accumulation appeared 1.6-fold higher in whole male turtle embryos. Since *MALAT1* is a ncRNA that shows dimorphic expression in the TSP we felt it was an excellent candidate for a gene involved in TSD so we used quantitative real-time PCR (qRT-PCR) to verify the pattern of expression suggested by the macroarray.

**Figure 4:**
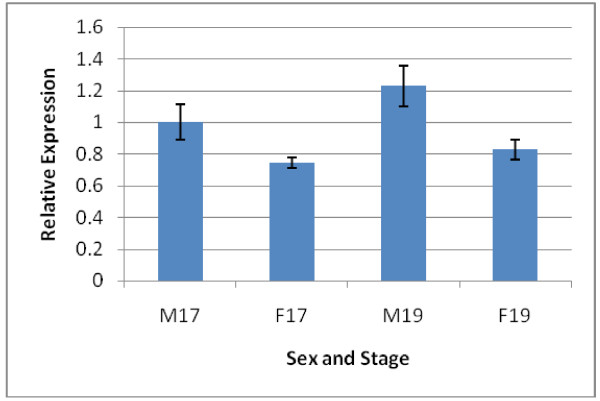
**Quantitative RT-PCR showing sexual dimorphic expression at stage 17 and 19.** The expression of *MALAT1* (n = 5) consists of sex and stage (e.g. M17 = male, stage 17) and is relative to the control gene (*PP1*). The relative expression for M17 was set as 1 to allow easier comparison between groups. Gene expression was analysed using two-tailed *t*-test to examine differences between sexes at the same stage (stage 17, *P* = 0.04 and stage 19, *P* = 0.01) and between stages of the same sex (male, *P* = 0.18 and female, *P* = 0.24).

We used qRT-PCR to examine *MALAT1* RNA accumulation because it represents a rigorous test of differential expression. *MALAT1* RNA accumulation was examined independently for multiple individuals (n = 5) and the two stages during the TSP (stages 17 and 19) rather than using pooled samples. This analysis revealed a slight but significant sexual dimorphism (about 1.4-fold higher in males) in the amount of *MALAT1* RNA during both stages we examined (Figure 4). *MALAT1* RNA expression also shows a modest increase as development progresses from stage 17 to stage 19 in both males and females. These observations are consistent with the hypothesis that *MALAT1* plays a role in turtle TSD.

## Conclusions

Here we reported a survey of genes identified based upon their patterns of mRNA accumulation during embryonic development in the Red-eared slider turtle. We used a non-ortholog based strategy and identified four genes that exhibited increased mRNA expression as development proceeded from stage 14 to stage 17, a set of genes that responded to E_2_ exposure, and two genes (*MALAT1* and *C16ORF62*) that show greater accumulation at the male producing temperature than at the female producing temperature. This survey focused on changes in mRNA accumulation in whole embryos. Thus, it remains possible that some or all of these genes exhibit even more strongly dimorphic expression in specific tissues (e.g., the developing gonad or brain). Moreover, the genes we identified are likely to be significant since screening for differential expression at the whole embryo level is expected to be a conservative way to examine gene expression during development.

*MMP*s (matrix metalloproteinases) are involved in the breakdown of extracellular matrices in physiological processes, including cancer [71]. *MMP2* is sexually dimorphic in developing male mice because it functions as a paracrine death factor in Müllerian duct regression downstream of the *MIS* cascade [50]. In addition, Kim et al. 2008 [72] found *MMP2* to be sexually dimorphic and regulated by testosterone in songbirds in relation to the vocal control center during adult neurogenesis. Furthermore, estrogen affects the MMP pathway in humans by increasing MMP2 enzymatic activity [73]. It is unclear if the increase in MMP2 is through an increase in mRNA accumulation or through other mechanisms, such as binding affinity changes. Though *MMP2* was not found to be sexually dimorphic in turtles it was found to be inhibited by E_2_, the opposite of the regulation in mammals but potentially similar to birds [72]. Together with the prior knowledge of its involvement in mammalian and avian development, *MMP2* is a novel candidate gene for development in the turtle.

ncRNAs are believed to play a large number of biological roles (reviewed in [74]), but their role in development remains poorly characterized [75]. Although there is some evidence that ncRNAs have roles in sexual development in both mammals [75] and birds [76], this is the first evidence that a ncRNA may have a role in sexual development for an organism with TSD. This hypothesis is corroborated by the fact that *MALAT1* exhibits differential expression in mammalian gonads (expression is higher in adult ovaries than in testes). However, the pattern of sexual dimorphism reported for mammals is distinct from that evident in turtles (where the RNA accumulation is higher in male embryos than in female embryos). Our findings highlight the importance of examining ncRNAs when investigating vertebrate development in general and sexual development specifically.

Little is known about the genes involved in turtle development, including the processes related to TSD, regardless of whether they are protein-coding genes or ncRNAs. Much of the information available is focused on the examination of the turtle orthologs of genes involved in mammalian development. The limitations on this type of an examination are exemplified by our novel results of two genes (*MALAT1* and *C16ORF62*) that show sexual dimorphism during TSP. These two types of genes are novel to turtle sexual development because one is a ncRNA (*MALAT1*), not typically found in sexual differentiation in other vertebrates, and the other (*C16ORF62*) is of unknown function. Our results highlight the need to diverge from focusing only on protein-coding genes when looking for developmental genes and to expand into the more diverse world of RNA in general, specifically including ncRNAs.

## Methods

### Incubation and experimental manipulations

Freshly laid *Trachemys scripta* eggs (500) were purchased from Kliebert Turtle Farms in Hammond, Louisiana in 2004 and 2006. They were kept at room temperature for less than 48 hours until they were established as viable by candling. Those viable were randomly separated equally into four experimental groups in containers with moistened vermiculite (1:1 vermiculite to water). The experimental groups were the female producing temperature of 31°C (hereafter called female), the male producing temperature of 26°C (hereafter called male), 26°C painted with exogenous estradiol-17β (E_2_) in 1 μg/μL in 95% ethanol (non-denatured) (phenotypically female; hereafter called E_2_), and 26°C painted with exogenous 95% ethanol (non-denatured) as the vehicle control (phenotypically male; hereafter called vehicle). Application of E_2_ and vehicle occurred at stage 14. The egg boxes were rotated daily within the incubators and we checked a random selection of eggs periodically to determine their developmental stage using the staging guidelines suggested by Yntema [77]. The temperature was monitored daily with HOBO data loggers and in-incubator thermometers. Sex was determined for each experimental group through a visual inspection by two independent researchers at hatching (gonads are visually distinct at hatching but not before) of 10 embryos per experimental group for relevant gross anatomy. All experiments complied with the appropriate ethical guidelines.

### Isolation of RNA

Whole embryos were taken between stages 17 and 20 from each experimental group and quickly frozen in liquid nitrogen and stored at −80°C. A subset of whole embryos was also collected at 0, 6, and 24 hours after E_2_ and vehicle application at stage 14. An average of 5 embryos was collected per stage and experimental group. Conducting the E_2_ time trial at stage 14 before the TSP removes E_2_ effects within gonadal differentiation and leaves just those from the trial. Total RNA was extracted from each embryo by homogenization in Tri-Reagent (TRIzol, Sigma USA), followed by extraction in chloroform, and precipitation in isopropanol according to Sambrook and Russell [78]. Total RNA yield and quality were assessed with the ND-1000 Nanodrop spectrophotometer (NanoDrop Technologies, Thermo Fisher Scientific, Wilmington, DE 19810, USA), and the integrity was verified by electrophoresis on a 1% agarose gel.

### Suppression subtractive hybridization (SSH)

Three libraries were selectively induced for female against male, male against female, and E_2_ against vehicle. Testers and drivers were made from pooled RNA from stages 17–20 (two individuals per stage) from each experimental group from 2004 (stated above). cDNA synthesis was performed with the BD SMART™ PCR cDNA Synthesis Kit (Clontech, Mountain View, CA) according to the manufacturer’s protocol. Three subtraction libraries were constructed with the Clontech PCR–Select^TM^ cDNA Subtraction Kit (Clontech, Mountain View, CA) according to the manufacturer’s protocol except a PEG (PolyEthylene Glycol) precipitation followed by an ethanol wash was used to purify the PCR products after cDNA synthesis instead of the column chromatography. The resulting cDNA was ligated into a pGEM-T Easy vector and transformed into *E. cloni*® 10 G electrocompetent cells (Lucigen) by the manufacturer’s protocol. Individual colonies were picked and stored in 96-well plates with 50% glycerol at −80°C. Plasmid inserts were purified using a modified 96-well Perfectprep® Plasmid protocol (5Prime, Gaithersburg, MD), according to Sambrook and Russell [78] or a TempliPhi Amplification kit (as recommended by manufacturer; GE Healthcare). Single-pass sequencing was conducted on an ABI Prism™ 3100-Avant genetic analyzer (PE Applied Biosystems) using the ABI BigDye® Terminator v.3.1 chemistry.

### Analysis of SSH results

Sequences from the libraries with redundancy were aligned and all sequences (both individual and aligned) were edited in Sequencher™ 4.1 (Gene Codes Corp.). Sequences were put into FASTA format and run in GOanna from the AgBase v.2.0 database to determine the top Blast hit for each sequence and to simultaneously determine GO terms for each hit [79]. GOanna uses BLASTX (determines gene products from sequences) therefore any sequences that did not have a hit were run through BLASTN (determines all aspects of RNA transcripts including untranslated regions and non-protein coding RNAs) on the NCBI server [57].

GeneMerge categorized the human homologs of genes that resulted from all three SHH libraries with over-represented GO (Gene Ontology) terms from the biological processes category given a human background set of genes [47]. The significance cut-off was set at *P* < 0.05.

The genes that have human homologs from the SSH libraries were clustered into functionally related groups within the subset of biological processes by DAVID v.6.7 [48,49]. The DAVID tool for functional annotation clustering uses GO terms and the term enrichment score was used at high stringency (based on *P* < 0.05).

### Macroarray preparation and analyses

Three hundred and seventy four clones (including 322 known clones [those with BLAST hits] and 61 unknown clones) that were obtained from all three SSH libraries (discussed above) were spotted onto membranes (Pall Biodyne B Nylon, Nunc) using 100 nanoliter pins on a Biomek 2000 (Beckman Coulter, USA). Positive controls (*Arabidopsis thaliana RCA* [X14212], *CAB* [X56062], and *RBCL* [U91966]) and negative controls were also spotted onto the membranes. All samples were spotted in duplicate with four replicates per experimental group (female and male). Total RNA was collected (as stated above) from 2 embryos from stage 17 and 2 embryos from stage 19. All collected embryos from the male experimental group in 2004 were pooled as well as for the female experimental group. Pooled total RNA was mixed with control cDNAs and then reverse transcribed before labelling with α^33^P-dATP as described in Blum et al. [80]. After hybridization [80], the membranes were rinsed and exposed to a phosphor imager and scanned using a Molecular Devices Typhoon Scanner.

Signal intensities were quantified using ImageQuant 5.1 (Amersham Biosciences UK Limited, Amersham Place Little Chalfont Buckinghamshire England) and intensity differences were calculated as described by Helbing et al. [81]. Briefly, image data were converted to a standard 8-bit TIFF file before accounting for signal saturation. Positive controls used to standardize across arrays for hybridization efficiency were chosen based on their coefficient of variation (cv) across all 8 arrays (the cv did not exceed 0.2). Each array was normalized to the geometric mean for all positive controls chosen for that array [82]. The non-signal background was determined using the median intensity value plus one standard deviation for the blank positions and negative controls, and the maximum value for all arrays was set as the “no signal” value. All values across all arrays equal to or below that number is considered zero. After normalizing across arrays and discarding values equal to or below the floor value, the average of the duplicates within arrays, the estimated standard deviation across replicate arrays, the median across replicate arrays, and the fold change between treatment groups were calculated. If the standard deviation across replicates was greater than or equal to 2 then those spots were not reliable for further examination. Based upon Helbing et al. [81], we considered a 1.5-fold difference in signal intensity relative to control treatments as sufficient in the array experiments based on the detection limitations of the cDNA array analyses. Those genes that exhibited differential expression were used for further examination.

### Semi-quantitative PCR preparation and analysis

Embryos were collected and total RNA was extracted as stated above from the 31°C experimental group at stage 17, the 26°C experimental group at stage 17, and from the E_2_ time trial experimental group at stage 14 from 2006. Four embryos from each group were pooled and cDNA was made using Invitrogen’s Superscript III Reverse Transcription kit (Invitrogen, USA) following the manufacturer’s instructions.

Pilot experiments were conducted to determine optimum PCR conditions for the candidate genes and a control gene (*PP1*, [12]). PCR primers were designed using Primer3 [83] and a list of sequences and PCR conditions after pilot experiments can be found in Table 3. Controls were systematically run in each set of semi-quantitative assays: 1) a cDNA positive control for a known sample; 2) a PCR positive control; and 3) a negative control. An internal exogenous standard (cDNA synthesis with no reverse transcriptase) was also run separately for each cDNA mixture. PCR products were loaded onto a 1.5% TBE gel with ethidium bromide and a 1 kbp DNA ladder molecular weight marker (Minnesota Molecular) and electrophoresed at 90 V for 45 minutes.

**Table 3:** Semi-quantitative and quantitative real-time PCR primers, and optimal conditions for semi-quantitative PCR

**Gene symbol**	**Primer name**	**Primer Sequence**	**Annealing Temperature (°C)**	**MgCl**_**2**_Concentration (mM)*	**Cycle Number**
**Cct3**	Cct3 F	GGATGCCTAAAATTAGCCTCCTA	62.5	1.5	30
	Cct3 R	GAAGCTACGGCAAATGATGG			
**Malat1§**	Malat1 F	GTACGCGGGCAGACTAACAC	57.1	1.5	36
	Malat1 R	TGCGTCTAGACACCACAACC			
**C16orf62**	C16orf62 F	CGGCCGAGGTACAAATTAAG	58.3	2.5	36
	C16orf62 R	TGCAAGTGCATTATGGAAGC			
**Mmp2**	Mmp2 F	ATGAAGAAGCCCCGCTGTGGTAATCC	62.5	4.5	27
	Mmp2 R	AAAGGCATCGTCTACTGTTTCGGAGTCC			
**Nf1a**	Nfib F	AAACACACTGCGTCAAGTGC	61.4	1.5	24
	Nfib R	CTTGCCCTGGATAGCGATTA			
**Notch2**	Notch2 F	TATTTCTGTGGCTGCCTGGA	62.5	1.5	36
	Notch2 R	GGGACAGGGACCTTTGTTGT			
**Pp1§**	Pp1 F	ACCTCTTCCTGGGCGACTAT	62.5	1.5	27
	Pp1 R	TGATGTTGTAGCGCCTCTTG			

*total concentration, including any MgCl2 in Taq buffer.

§Same primers used for qRT-PCR but at 60°C annealing temperature and 40 cycles.

Analysis of gel images was conducted using ImageJ [84]. Each experimental gene was standardized to the control gene, *PP1*[12], and then normalized to the female group.

### Quantitative Real-time PCR (qRT-PCR) preparation and analysis

Five whole embryos were collected from stage 17 and 5 from stage 19 from two experimental groups, male and female, and total RNA was extracted as stated above. cDNA was generated using ImProm-II™ Reverse Transcriptase and random primers following the manufacturer’s instructions. Relative gene expression levels were quantified using an ABI StepOnePlus™ Real-time PCR cycler (STepOne™ Software v2.1) with the following cycling parameters: initial denaturing for 10 min at 95°C, followed by 40 cycles of 35 s at 95°C, 30 s at 60°C, and 30 s at 72°C. The final cycle was followed by a melting curve analysis to verify the amplification of a single product in each well. Specificities of all primer pairs were also verified by sequencing PCR products. Repeating the above procedures on RNA samples (prior to reverse transcription) verified that no products were amplified from contaminating genomic DNA. All samples were run in duplicate and included 3.75 μL of a 1:100 diluted sample, 1 μM of each primer, and 2x SYBR Green Master Mix (Applied Biosystems) in a total of 15 μL. PCR efficiencies were calculated from a gene-specific standard curve from a 10-fold dilution series. Relative transcript abundance was normalized to the expression of *PP1* by using the relative standard curve method [85]. To determine if expression differed between experimental groups a two-tailed Student’s *t*-test and a standard error analysis were performed. Primers used to assay gene expression were designed using Primer 3 [85] and Amplify [86] (Table 3).

## Competing interests

The authors declare they have no competing interests.

## Authors’ contributions

JLC carried out all data collection, participated in the experimental design and drafted the manuscript. ELB participated in the experimental design, bioinformatic analyses, and helped to draft the manuscript. All authors read and approved the final manuscript.

## Supplementary Material

Additional file 1 **Homology searches for subtraction libraries.** Homology searches for sequences from all 3 libraries. The table includes clone identifier, match, accession number, e-value, library, and redundancy. Click here for file

Additional file 2 **GeneMerge for biological processes.** GeneMerge determined over-represented GO terms for my gene set given a human gene set background. The cut-off was set to P < 0.05. Click here for file

Additional file 3 **DAVID functional annotation clustering.** The DAVID clustering tool was used to determine functionally related groupings based on my gene set from the SSH libraries. The term enrichment was set at high stringency. Click here for file
